# Shedding Light on Microvascular Inflammation: Understanding Outcomes, But What Sparks the Flame?

**DOI:** 10.3389/ti.2024.14032

**Published:** 2024-11-26

**Authors:** Louise Benning, Oriol Bestard

**Affiliations:** ^1^ Department of Nephrology, Heidelberg University Hospital, Heidelberg, Germany; ^2^ Department of Nephrology and Kidney Transplantation, Vall d’Hebrón University Hospital, Barcelona, Spain

**Keywords:** kidney transplantation, antibody-mediated rejection, biomarkers, microvascular inflammation, targeted therapies

In their recent article, Drs. Sablik and Sannier, along with more than 40 international collaborators, examined the impact of different microvascular inflammation (MVI) phenotypes on allograft outcomes by analyzing a total of 16,293 allograft biopsies from 6,798 patients across over 30 transplant centers in Europe and North America [1]. Clinical and pathological data was used to reclassify biopsy specimens according to the 2022 BANFF Classification of Renal Allograft Pathology now including the two new diagnostic categories of probable antibody-mediated rejection (ABMR) and MVI without evidence of an antibody-mediated response [2]. The newly identified phenotypes were present in 788 specimens, of which 641 were previously categorized as no rejection by the BANFF 2019 classification [3].

In terms of graft loss, patients with *ABMR* and those with the newly considered histopathological phenotype, *MVI without antibody-mediated response* (DSA-/C4d-) showed an increased risk of 2.7 (95% 2.2–3.3) and 2.1 (95% CI 1.5–3.1), respectively, when compared to non-rejection cases, whereas patients with the diagnosis of *probable ABMR* did not show an increased risk through the following 5 years after biopsy (Hazard Ratio [HR] of 1.3; 95% CI 0.8–2.1). In terms of progression to ABMR, patients with DSA-/C4d- MVI and those with probable ABMR showed a comparable risk of progression, with an intermediate cumulative incidence of ABMR during follow-up, positioned between patients without MVI and those with active ABMR (subdistribution HRs of 0.4 [95% CI, 0.3–0.5] and 0.7 [95% CI, 0.4–1.2], respectively). Finally, when analyzing the risk of progression to transplant glomerulopathy, the DSA-/C4d- MVI group showed, once more, a similar risk to that in the probable ABMR group, again falling in between the risks seen in those without MVI and those with active ABMR.

In short, this extensive population-based study, utilizing a remarkable dataset of allograft biopsies, compellingly demonstrates the importance of recognizing MVI as distinct histopathological phenotypes that associate with different disease progression and allograft failure. Notably, patients with MVI fulfilling the complete ABMR diagnosis display worse graft outcome, aligning with prior studies suggesting that patients with MVI and incomplete humoral phenotypes display better outcomes than those with full ABMR but worse than patients without rejection [4–6]. Crucially, the study emphasizes the necessity for broader acknowledgment of the MVI phenotypes in clinical practice, which have frequently been overlooked until recently. The discussed findings highlight the advantages of the 2022 BANFF classification in capturing the clinical, histological, and prognostic diversity of MVI over the previous version, thus establishing a foundation for standardizing future trials aimed at elucidating the immunological mechanisms behind these distinct phenotypes and potentially guiding tailored therapeutic strategies. Interestingly, the authors further propose that their findings may extend to other solid-organ transplants, where MVI is also a key diagnostic feature of ABMR, indicating possible similarities in pathophysiological processes that merit further study.

Authors are to be commended for their collaborative effort in assembling this substantial dataset to investigate the newly defined BANFF phenotypes in relation to the advent of distinct allograft outcomes. Nonetheless, the precise pathophysiological mechanisms underlying the development of these newly considered histopathological phenotypes, and especially MVI without evidence of an antibody-response (DSA-/C4d-) still remain elusive, leaving the question of what truly sets the spark for MVI. This is of paramount importance as the identification of main effector mechanisms orchestrating such specific graft injuries would allow to consistently design guided therapeutic strategies within interventional clinical trials. Indeed, a clear example underscoring such endeavor was delineated already in 2001, when the diagnostic feature of ABMR was first incorporated into the Banff classification by including the basic histopathological lesions of MVI and key immunological parameters such as serum DSA or C4d deposition [7], with an expansion of the histopathological ABMR criteria later in 2013 to include endarteritis, when concomitantly found in presence of serum DSA [8]. While the causality link between the two features may not strictly be confirmed, the strong associations described between such specific allograft lesions and the presence of DSA, the downstream effector mechanism of an anti-donor B-cell alloimmune response, has provided the solidest basis for this histological diagnosis. Notably, advances in molecular transcriptomics have helped to further refine distinct histopathological features, especially T-cell mediated rejection (TCMR) and ABMR, thus ultimately allowing reclassification of allograft lesions not fully captured with the conventional light microscope [9–13]. Nevertheless, while some recent works have shown overlapping transcriptional signatures between ABMR and DSA-/C4d- MVI, suggesting a common ethiopathological origin [14, 15], it may be argued that such common gene perturbation merely illustrates the similar cellular infiltrate composition, rather than the mechanisms driving its development. Notably, growing evidence suggests that DSA-/C4d- MVI may be more closely linked to an innate immune response, with natural killer (NK) cell–driven allorecognition potentially playing a key role in allograft injury [16–22]. Yet, the precise role of NK cells in MVI remains unclear [16], as they constitute only a small portion of the inflammatory infiltrate in MVI, which seems otherwise largely dominated by macrophages and T-cells [22–24]. Indeed, recent multi-omic profiling has shown a notable T-cell presence and activity, suggesting a T-cell effector dominant phenotype [25]. It is also plausible that other innate immune effector mechanisms, including myeloid-and monocyte-driven allorecognition could lead to similar histological/molecular pictures [26]. Importantly, it is highly likely that these diverse alloimmune effector mechanisms are not mutually exclusive but may, in fact, rather interconnect in complex ways [16].

Additionally, current clinical trials are exploring various blood- and urine-biomarkers indicative of graft injury, frequently caused by rejection, with donor-derived cell-free DNA (dd-cfDNA) emerging as particularly promising for differentiating microvascular injury in ABMR [27–38]. Consequently, dd-cfDNA has already been cleverly implemented into recent trials targeting ABMR. For instance, treatment with the anti-IL6 monoclonal antibody clazakizumab did not result in significant changes in dd-cfDNA levels, indicating ongoing allograft injury [39], whereas, only recently, treatment with anti-CD38 monoclonal antibody felzartamab demonstrated notable changes in dd-cfDNA, suggesting a beneficial therapeutic effect with the apparent resolution of injury [40]. Notably, while biomarkers like dd-cfDNA signal graft injury, they provide only limited insights into the underlying mechanisms driving graft damage [41]. Ideally, biomarkers would also reflect lesion pathophysiology or track alloimmune responses, allowing a more comprehensive understanding of the graft injury process. Thus, further research is needed to explore how biomarkers can aid in differentiating the various rejection phenotypes, understand rejection pathophysiology, assist in monitoring treatment responses, and be used to predict patient outcomes.

In consequence, as we now acknowledge novel kidney allograft rejection phenotypes and their different associated outcomes, it is essential to deepen our understanding of the main mechanisms driving these histopathological lesions by means of exploring immunological biomarkers and functional diagnostic tools tracking alloimmune responses, beyond conventional histology and DSA measurements. These advancements, along with others, will then represent a significant step forward in personalized care to optimize patient and allograft outcomes (Figure 1).

**FIGURE 1:**
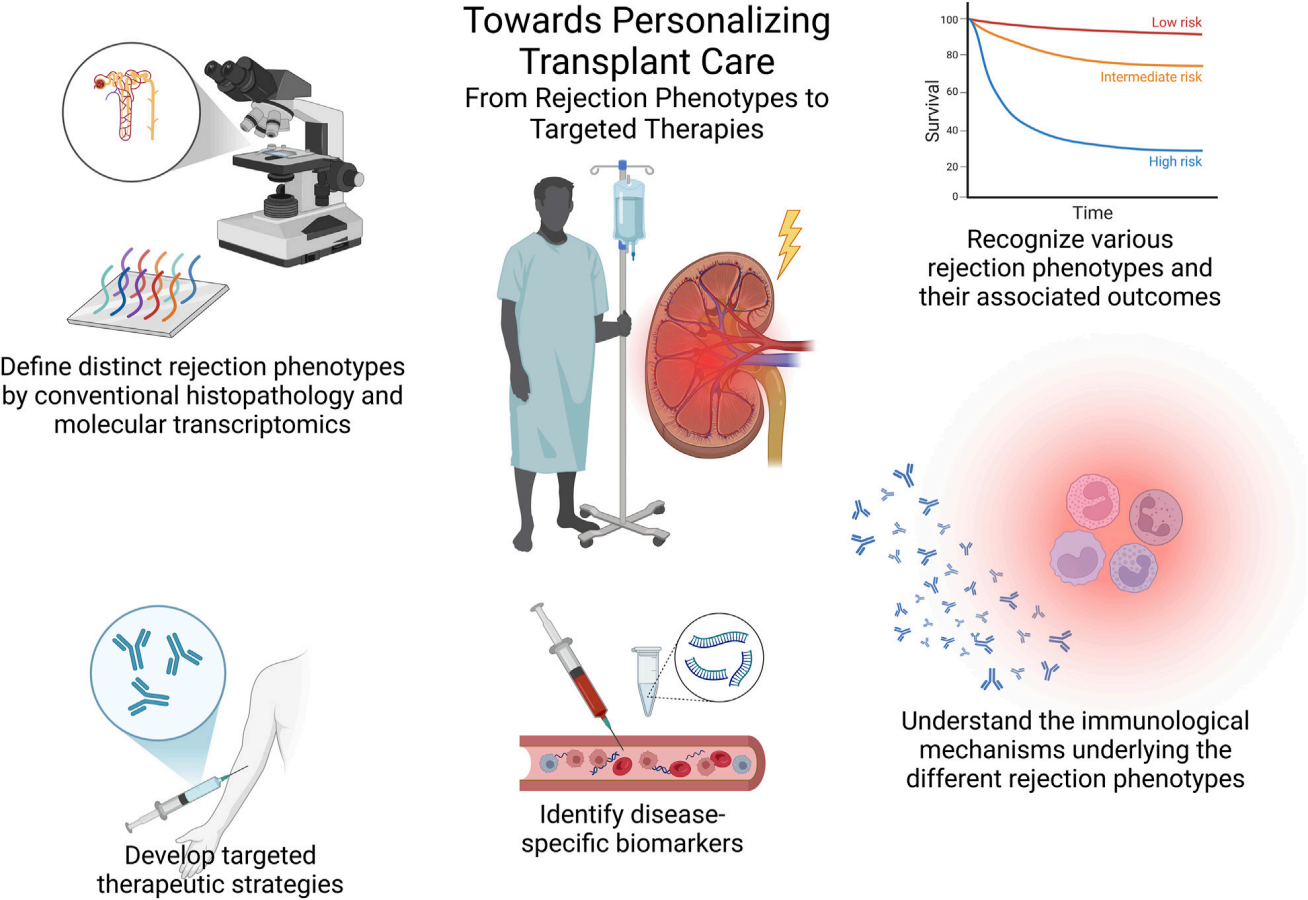
Towards personalizing transplant care – from rejection phenotypes to targeted therapies. This summary figure illustrates the essential steps toward personalized transplant care, including the classification of rejection phenotypes through conventional histopathology and molecular tools, understanding associated outcomes, exploring underlying immunological mechanisms, identifying disease-specific biomarkers, and ultimately developing targeted therapies based on these insights.

## Data Availability

The original contributions presented in the study are included in the article/supplementary material, further inquiries can be directed to the corresponding author.
